# Eco-Efficiency and Its Drivers in Tourism Sectors with Respect to Carbon Emissions from the Supply Chain: An Integrated EEIO and DEA Approach

**DOI:** 10.3390/ijerph19116951

**Published:** 2022-06-06

**Authors:** Bing Xia, Suocheng Dong, Zehong Li, Minyan Zhao, Dongqi Sun, Wenbiao Zhang, Yu Li

**Affiliations:** 1 Institute of Geographic Sciences and Natural Resources Research, Chinese Academy of Sciences, Beijing 100101, China; xiab.16b@igsnrr.ac.cn (B.X.); lizehong@igsnrr.ac.cn (Z.L.); sundq@igsnrr.ac.cn (D.S.); 2 College of Resources and Environment, University of Chinese Academy of Sciences, Beijing 100049, China; 3 Institute of Tibetan Plateau Research, Chinese Academy of Sciences, Beijing 100101, China; my.z@yeah.net; 4 Beijing Academy of Social Sciences, 33 North Fourth Ring Middle Road, Chaoyang District, Beijing 100101, China; zhangwenbiaozwb@163.com

**Keywords:** eco-efficiency, tourism sector, carbon emissions, supply chain, environmentally extended input–output analysis (EEIO), data envelopment analysis (DEA)

## Abstract

Eco-efficiency analysis can provide useful information about sustainability in the tourism industry, which has an important role in both global economy recovery and Sustainable Development Goals (SDGs), generating considerable indirect carbon emissions with respect to the supply chain due to its significant connections to other industries. This study, from the perspective of tourism sectors, including tourism hotels, travel agencies, and scenic spots, integrated the environmentally extended input–output analysis (EEIO) and data envelopment analysis (DEA) models to develop a research framework, analyzing the indirect carbon emissions of the tourism supply chain, evaluating eco-efficiency with respect to both direct carbon emissions and total carbon emissions (including direct and indirect parts), and exploring the driving factors of eco-efficiency of tourism sectors using Tobit regression models. This study took Gansu as a case, a province in China characterized by higher carbon intensity, an underdeveloped economy, and rapid tourism growth. The results demonstrate that (1) tourism hotels contribute the most carbon emissions in tourism sectors, especially indirectly due to the supply chain, with carbon emissions mainly resulting from the manufacturing of food and tobacco; (2) the eco-efficiency of tourism sectors in Gansu presents a U-shaped curve, which is consistent with Kuznets’ theory; and (3) energy technology is key to improving the eco-efficiency of tourism sectors. The research results provide a clear path for the reduction of carbon emissions and the improvement of eco-efficiency in Gansu tourism sectors. Against the backdrop of global climate change and the post-COVID-19 era, our research framework and findings provide a reference for similar regions and countries who are in urgent need of rapid tourism development to effect economic recovery.

## 1. Introduction

Facing a post-COVID-19 era, tourism, which contributes over 10% to global economic growth [1], may play a significant role in economic recovery. However, over the past several years, the tourism industry has seen an increase in the consumption of natural resources and energy [2] as well as significant increases in carbon emissions and the disposal of other types of waste [3]. Because the tourism industry has an extremely complex input–output relationship and involves a large number of intermediate input sectors on its supply chain [4], some scholars have begun to use the input–output method to evaluate carbon emissions in the supply chain of tourism [5,6]; furthermore, scholars have found that global carbon emissions, including indirect emissions from the supply chain, are four times higher than the direct carbon emissions of tourism [7]. At present, the input–output method based on supply chain is a popular tool in the evaluation of carbon emissions and carbon footprints, especially within economic sectors. The supply chain here refers to all the supply sectors of intermediate inputs needed in the production of goods and the provision of services [8]. For different tourism sectors, there is a wide range of related inputs, such as the intermediate input of tourism hotels, including the food and tobacco needed by catering services, the textile and furniture needed to provide accommodation services, and so on. Hence, assessing tourism carbon emissions from a supply chain perspective is necessary to provide a clear path for the reduction of carbon emissions against the backdrop of global climate change [9]. Some scholars have begun to focus on a more comprehensive accounting of carbon emissions. Sun [10] presents an environmentally extended input–output (EEIO) model to assess the distribution of tourism’s economic and carbon emission effects on bilateral travel between Taiwan and Japan. However, because the tourism industry plays a vital role in achieving all 17 SDGs, specifically, ending poverty (SDG 1), decent work and economic growth (SDG 8) [11], and responsible consumption and protection (SDG 12) [12], only assessing its carbon emissions is inherently biased [13,14], especially due to the urgent demand for economic recovery in the post-COVID-19 era. In sum, facing economic recovery and global carbon emission mitigation pressure, the tourism industry needs to focus on seeking more precise emission reduction objectives—tracking indirect carbon emission sources by using the input–output method, and, moreover, exploring driving factors that will improve the comprehensive benefits of the tourism economy and carbon emission mitigation. Therefore, it is important to comprehensively assess tourism’s dual impacts both on economic growth and global climate change in order to find a path to sustainable development.

Eco-efficiency analysis, as proposed by the World Business Council for Sustainable Development (WBCSD) [15], can provide useful information about the sustainability of the tourism industry. Eco-efficiency in the tourism industry is defined as determining the environmental ecology impact per unit of tourism value and developing a carbon dioxide assessment method [16]. The core concept of eco-efficiency is an optimization scheme, which is a path to achieving economic growth while mitigating the environmental impact of tourism [17]. Against the backdrop of global climate change, carbon emissions are usually selected as the environmental ecology indicator in order to analyze tourism eco-efficiency, combined with data envelopment analysis (DEA), which can include multi-input and -output indicators [17,18]. Pan (2021) calculated tourism carbon emission efficiency based on the super-efficiency Slacks Based Measure (SBM-DEA), which is defined as a compound system that consists of tourism carbon emissions, tourism economic development, and tourism regional innovation [19]. However, there is little existing research integrating EEIO and DEA to analyze the eco-efficiency of tourism using direct and indirect carbon emissions. Zha (2020) used the EEIO method to calculate the carbon emissions of tourism in China and used data envelopment analysis (DEA) to examine the sources of change in tourism CO_2_ emissions [20]. However, in Zha’s paper, tourism was taken as a whole industry, despite the fact that the carbon emission process and the economic operation laws of different tourism sectors, such as hotels, tourism agencies, and scenic spots, are significantly different.

Based on the above tourism development background and relevant research progress, this research tries to fill the knowledge gaps with the following hypotheses. The first is in regard to Gansu province, where tourism is developing rapidly: What are the time trajectories and development differences between direct and indirect carbon emissions within different tourism sectors? The second considers the input–output relationship between different tourism sectors and other relevant economic sectors: What are the main sources of indirect carbon emissions? The third is considers the two scenarios of direct carbon emissions and total carbon emissions, including indirect carbon emissions: What is the evolutionary trajectory of eco-efficiency in different tourism sectors? The fourth is: What are the driving factors of eco-efficiency in the tourism sector under different carbon emission scenarios? The innovations of this research are as follows: the first is integrating EEIO and DEA in order to analyze carbon emissions, including direct and indirect parts of the supply chain in different tourism sectors, and to fully consider their economic growth; the second is analyzing from the perspective of specific tourism sectors, including travel agencies, scenic spots, and hotels, which supply the most tourist production and services in China; the third is putting forward more targeted suggestions with respect to the main, indirect carbon emission contributors in the supply chain and the driving forces of eco-efficiency in specific tourism sectors.

This research built a widely used research framework for the comparative evaluation of carbon emissions and eco-efficiency in different sectors of tourism. It did so in order to apply scientific guidance to the targeting of carbon emission mitigation within the supply chain of various tourism sectors, while also keeping the economy growing as much as possible. The rest of this paper is organized as follows: Section 2 reviews the relevant literature and constructs the research framework, including the research boundary and roadmap and the study area; Section 3 discusses the research methods, including the model and data source; Section 4 presents the research findings, including results and analysis; Section 5 presents discussions and implementations; and conclusions are presented in Section 6.

## 2. Literature Review and Research Framework

### 2.1. Literature for Carbon Emissions Evaluation of Tourism

Against the backdrop of global climate change, carbon emissions from the tourism industry have become a hot topic in the increasing number of countries that are experiencing rapid tourism development [21,22], such as China [23], New Zealand [9], Portugal [24], Spain [25,26], Italy [27], Turkey [28], Brazil [29], and so on. Carbon emissions, including within direct and indirect parts of the supply chain, are a topic of discussion among many scholars, who sometimes also include carbon footprint within this subject by incorporating direct and indirect domestic and imported virtual carbon, which are required to satisfy the demand for products by different tourism consumers [30]. Carbon footprint is a part of the ecological footprint concept, which quantifies the consumption and occupation of the ecological environment by human society [31]. In this research, the concept of carbon emission is selected for the comparative analysis of tourism eco-efficiency, including with respect to direct carbon emissions and complete carbon emissions.

Carbon emission assessments are mainly based on a “top-down” perspective on the energy supply side [32] and a “bottom-up” perspective on the energy consumption side [33]. Research on carbon emissions in the tourism industry using the “bottom-up” approach focuses on the consumption side and assesses emissions by studying the energy consumption coefficient per unit of the output of various transportation modes [34], travel hotels [35], and tourist activities [36]. However, as tourism development is closely related to a range of economic sectors, including transportation, trade, food and beverage, and wholesale and retail, only measuring carbon emissions by assessing consumption in tourism will not enable researchers to understand the real situation with regard to tourism’s impact on climate change. Furthermore, using this approach entails a large amount of work, and it is difficult to conduct a timeseries analysis.

As EEIO accounts for interindustry connections, and considering the characteristics of input–output tables, as well as multiple timeseries [37,38], EEIO is an important tool for the assessment of carbon emissions [39,40]. This method has even more advantages in regard to assessing emissions in the tourism industry, and it is able to present a complete scope of direct and indirect greenhouse gas emissions [41]. The approach covers direct emissions produced by tourism sectors and all aspects of indirect effects throughout the supply chain within and outside the destination country [42]. There are three generally agreed upon views within carbon emissions research among existing scholars. First, the carbon emissions of tourism, including indirect carbon emissions, are higher than what is known, a subject that requires attention. Second, although the carbon emissions of tourism are high, the industry brings great social and economic benefits; thus, it is necessary to explore the coordinated development model of tourism ecology and economy. Third, EEIO is considered to be an effective and comprehensive assessment method to measure carbon emissions in macro tourism sectors.

### 2.2. Literature for Eco-Efficiency Evaluation of Tourism

Separate carbon emission accounting of tourism sectors has limited policy support and practical guidance for the low-carbon development of the industry; e.g., studies have shown that holiday tourists have far higher carbon emissions than sightseeing tourists (due to increased accommodation emissions) [43], and another study showed that the carbon emissions of long-distance tourists are far higher than short-distance travelers due to increased traffic emissions [44]. However, from the perspective of the tourism economy, holiday tourists and long-distance tourists make more contributions to their destination’s economy than sightseeing tourists and short-distance tourists. Therefore, research on tourism eco-efficiency based on carbon emissions has expanded into the field of tourism carbon emissions, and, furthermore, has become the hot spot of tourism eco-efficiency research.

Gossling proposed a way to measure the eco-efficiency of tourism based on carbon emissions [16]. He used a single ratio method to compare the eco-efficiency of tourist destinations in France, Amsterdam in Denmark, Seychelles, Siena in Italy, and the Rocky Mountain National Park in the United States, and he found that there were great differences in carbon emissions efficiency between different sectors of tourism. Besides the single ratio method [45], the indicator method [46] and data envelopment analysis (DEA) method have been widely applied to measure eco-efficiency [47], especially the undesirable output model of a slack-based model (undesirable-SBM) [48].

In the post-COVID-19 era and against the backdrop of aggravating global climate change, scholars have found that building prosperous and resilient low-carbon tourism needs a tool that can estimate the balance between economic benefits and eco-environmental impact [49], such as eco-efficiency [9]. A reduction in tourism carbon emissions is necessary in consideration of tourism’s value to the economy and the SDGs, thus, formulate targeted policies will help coordinate economic growth and carbon emission reduction.

### 2.3. The Research Boundary of Carbon Emissions and the Eco-Efficiency of Tourism

Existing research on carbon emissions and the eco-efficiency of the tourism industry mainly treats the industry as a whole [7,16]. The tourism industry comprises sectors that provide various types of consumption to tourists [4]. Based on the definition of the World Tourism Organization of the United Nations (UNWTO), the tourism industry consists of five major sectors related to travel, including transportation, leisure and entertainment, accommodation, food and beverage, and travel agencies. The products and services supplied by each sector are significantly different. Thus, analyzing the carbon emissions and eco-efficiency of tourism as a whole makes it difficult to identify mechanisms for the reduction of carbon emissions and the improvement of eco-efficiency.

Scenic spots, hotels, and travel agencies—based on the operating and management data collected continuously by the statistics department of China’s government [50]—are separate sectors and highly representative of the production and service processes of the tourism industry: Scenic spots are a spatial aggregation form of tourism attractions where tourist activities and carbon emissions occur [51,52]; as a sector that provides packaged tourism products and services, travel agencies almost represent the entire consumption process in tourism [53]; and accommodation and food and beverage services usually represent the most energy consumption and carbon emissions in the tourism industry [54], if not including traffic.

The above three tourism sectors differ significantly from each other in terms of their services, the mode in which they provide services and operate businesses, and their carbon emission processes. As such, this article focuses on scenic spots, hotels, and travel agencies as the main tourism sectors, comparing and analyzing their carbon emissions and eco-efficiency, as well as the drivers of these three sectors in the comprehensive EEIO and DEA, in order to provide a reference and a research framework for tourism in similar regions.

### 2.4. The Research Framework

Based on the existing research, it has been found that direct carbon emission estimates obviously underestimate the carbon emissions of tourism. Input–output analysis can measure the carbon emissions of tourism, including the supply chain, and it can even overcome the heavy work of timeseries data due to bottom-up analysis. Furthermore, data envelopment analysis is a more comprehensive evaluation method of eco-efficiency, with multiple inputs and multiple outputs. In the post-COVID-19 era and against the backdrop of increasing global climate change, the study of carbon emissions in tourism is necessary to search for a coordinated development model, balancing carbon emissions and tourism’s economic benefits. Therefore, this article analyzes the eco-efficiency of tourism sectors with respect to direct carbon emissions and total carbon emissions, considering interindustry input–output relationships by integrating data envelopment analysis and input–output analysis. Moreover, considering the differences in economic operation laws and carbon emission paths among different sectors of tourism, this article focuses on the comparative analysis of direct carbon emissions and the indirect carbon emissions of tourism hotels, travel agencies, and scenic spots, as well as eco-efficiency and its drivers both with respect to direct and total carbon emissions.

**Figure 1:**
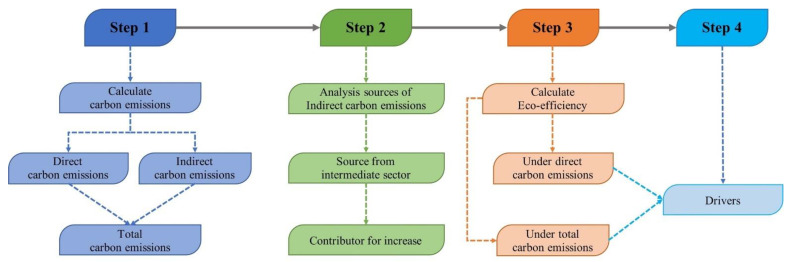
Research framework.

The specific research framework is as follows (Figure 1):

Step 1: To calculate the direct and total carbon emissions of the three tourism sectors through input–output analysis.
Step 2: To analyze the main sources of indirect carbon emissions in the three tourism sectors through input–output analysis.
Step 3: To estimate eco-efficiency with respect to direct and total carbon emissions in the three tourism sectors through the use of DEA.
Step 4: To reveal the main drivers of the three tourism sectors through the Tobit timeseries regression model.

### 2.5. Study Area

**Figure 2:**
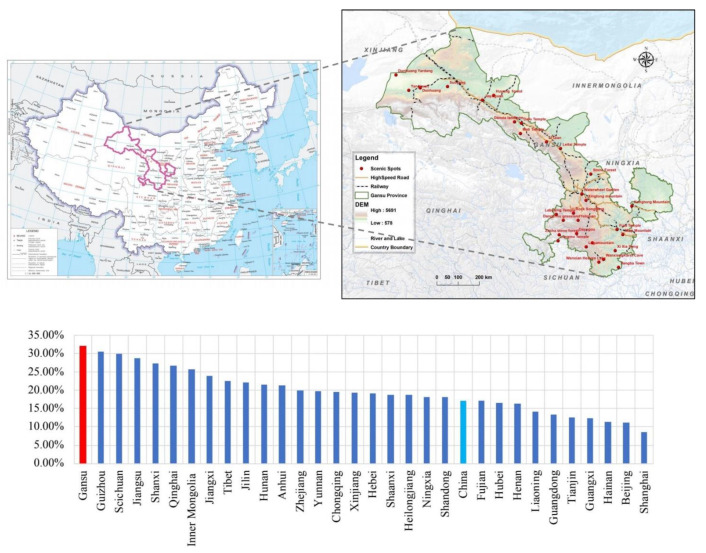
Study area and its economic growth with respect to the tourism industry from 1997 to 2016.

Gansu Province is located in northwest China, where the Mongolian Plateau meets the Qinghai–Tibet Plateau (Figure 2), and is vulnerable to climate change [55]. This area was the core of the ancient Silk Road. The marginalized, transitional geographic location bestows the province with abundant, diversified natural attractions and a cultural heritage that constitute significant, innate advantages for tourism development. In 2012, high-speed train services became available in Gansu, triggering a massive surge in tourism development. From 1997 to 2016, the average growth in total tourism revenue in the province ranked first in China (Figure 2). Tourism has become a new industry that drives regional economic growth and green development in Gansu, a province that traditionally relied on resources for its economy. Wang et al. (2019) estimated the carbon emissions of each province’s tourism industry between 2001 and 2016 and found that tourism in Gansu generated the highest carbon intensity level across China [56]. Therefore, as a typical province where the tourism industry is facing the dual pressures of economic growth and climate change, Gansu is illustrative for tourism sectors exploring a path to low-carbon, green development with respect to carbon emissions constraints. Herein, tourism hotels, travel agencies, and scenic spots in Gansu Province are taken as study subjects in order to be references to inform the low-carbon development of the tourism industry under the dual pressures of economic recovery and carbon emission reduction.

## 3. Methods

### 3.1. Calculation of Direct Carbon Emissions

The calculation of direct carbon emissions in tourism sectors is mainly based on the standards set by the Intergovernmental Panel on Climate Change (IPCC). Under these standards, emissions are calculated based on each sector’s revenue and energy emission coefficient, as follows:

(1) $$\eta^{th}=\frac{\sum_{k=1}^{r}\delta_{k}\times EC_{k}^{ac}}{l^{ac}}$$

(2) $$\eta^{ta}=\frac{\sum_{k=1}^{r}\delta_{k}\times EC_{k}^{os}}{l^{os}}$$

(3) $$\eta^{ts}=\frac{\sum_{k=1}^{r}\delta_{k}\times EC_{k}^{os}}{l^{os}}$$

where *η^th^, η^ta^* and *η^ts^* denote the energy emission coefficient of hotels, travel agencies, and scenic spots, respectively; *δ_k_* is the total consumption of energy *k* in Gansu Province (*k* = 1, 2,…*r*); $EC_{k}^{os}$ and $EC_{k}^{ac}$ represent the total consumption of energy *k* by other economic sectors and the food and beverage sector in Gansu, respectively; and *l^ac^* and *l^os^* denote the value added from other economic sectors and the food and beverage sector, respectively. Direct carbon emissions of hotels are calculated as follows:

(4) $$CE_{direct\;}^{th}=\mu \times \eta^{th}\times TR^{th}$$

(5) $$CE_{direct}^{ta}=\mu \times \eta^{ta}\times TR^{ta}$$

(6) $$CE_{direct}^{ts}=\mu \times \eta^{ts}\times TR^{ts}$$

where $CE_{direct\;}^{th}$, $CE_{direct}^{ta}$, and $CE_{direct}^{ts}$ represent the direct carbon emissions of hotels, travel agencies, and scenic spots, respectively, and $TR^{th}$, $TR^{ta}$, and $TR^{ts}$ denote the total revenue of hotels, travel agencies, and scenic spots, respectively.

### 3.2. Calculation of the Total Carbon Emissions of Tourism Sectors Based on EEIO

The structure of the input–output tables for Gansu Province for the years 1997, 2002, 2007, and 2012 is presented in Table 1. The second quadrant where the background is yellow and $z^{ij}$ is denoted represents intermediate inputs and outputs; the first quadrant where the background is blue and $f^{i}$ is denoted represents final use; and the third quadrant where the background is green $l^{j}$ is denoted represents value added. The column in red where $z^{1j}$ to $z^{nj}$ are denoted represents the supply chain of sector *j* (S*j*). Specifically, the input–output table includes *n* production sectors; $f^{i}$ denotes the final use of sector *i*; $X^{i}$ denotes the total output of sector *i*; $l^{j}$ denotes the value added of sector *j*; and $Y^{j}$ denotes the total input of sector *j.*

Within the study period, hotels, travel agencies, and scenic spots belong to different sectors in the input–output table. The proportions that the operating revenues of the above key sectors account for in final use are used to calculate the intermediate use of other sectors using these three sectors, as well as the intermediate input of these three sectors into other sectors. Tourism sector supply chains are represented by the column vectors $z^{i\sim th}$, $z^{i\sim ta}$, and $z^{i\sim ts}$, and can be calculated as follows:

(7) $$z^{i\sim th}=\frac{TR^{th}}{f^{ac}}z^{i\sim ac}$$

(8) $$z^{i\sim ta}=\frac{TR^{ta}}{f^{os}}z^{i\sim os}$$

(9) $$z^{i\sim ts}=\frac{TR^{ts}}{f^{os}}z^{i\sim os}$$

where $z^{i\sim th}$, $z^{i\sim ta}$, $\mathrm{and}\;z^{i\sim ts}$ represent the intermediate use of sector *j* by sector *i* in hotels, travel agencies, and scenic spots, respectively; $z^{i\sim ac}$ denotes the intermediate use of sector *i* by the food and beverage sector, to which hotels belong in the input–output table; $z^{i\sim os}$ denotes the intermediate use of sector *i* by “other sectors,” to which travel agencies and scenic spots belong in the input–output table; and $f^{ac}$ and $f^{os}$ denote final use of the food and beverage sector and “other sectors.” The intermediate inputs made by hotels, travel agencies, and scenic spots into sector *j*, and the value added for each sector is obtained in the same manner. The tourism supply chain is column vector $z^{i\sim th}$.

Data for input–output tables are only prepared every five years. To obtain continuous data, based on total carbon emissions calculations, the existing input–output tables were consolidated in accordance with the classification of value added for industries prepared by the National Bureau of Statistics of China.

Based on the input–output tables, the relationship between the total economic output and the total amount of final use can be derived:

(10) $$X={\left(I-A\right)}^{-1}Y$$

where *X* denotes the total output matrix of all sectors; *Y* denotes the final use matrix of all sectors; *A* denotes the direct consumption coefficient matrix of all sectors; and ${\left(I-A\right)}^{-1}$ is the Leontief inverse matrix.

By replacing *Y* with the value added matrix denoted by *L* and transposing the Leontief inverse matrix, the right side of the equation represents the total input for producing a product.

(11) $$X={\left[{\left(I-A\right)}^{-1}\right]}^{T}L$$

Let *L* be the value added matrix of all sectors in a given year; then, X is a 1 × *n′* matrix, where *n′* denotes the total number of sectors in the input–output tables. Each of the three tourism sectors—tourism hotels, travel agencies, and scenic spots—belongs to one line in the equation and is denoted by $X_{t}^{th}$, $X_{t}^{ta}$, and $X_{t}^{ts}$, respectively. Total carbon emissions, including indirect emissions by star-rated hotels, travel agencies, and scenic spots, which are denoted by $CE_{total\;}^{th}$, $CE_{total}^{ta}$, and $CE_{total}^{ts}$, respectively, are calculated as follows:

(12) $$CE_{total\;}^{th}=\mu \times \eta^{th}\times X^{th}$$

(13) $$CE_{total}^{ta}=\mu \times \eta^{ta}\times X^{ta}$$

(14) $$CE_{total}^{ts}=\mu \times \eta^{ts}\times X^{ts}$$

### 3.3. Calculation of Indirect Carbon Emissions of Tourism Sectors

According to the input–output tables for 1997 and 2012, the indirect carbon emissions of each sector from other supply chain sectors are calculated according to input proportion. The indirect carbon emissions of each sector are calculated according to the input proportion.

(15) $$CE^{i\sim th}=(CE_{total\;}^{th}-CE_{direct)}^{th}\times \frac{z^{i\sim th}}{l^{j}}$$

(16) $$CE^{i\sim ta}=(CE_{total\;}^{ta}-CE_{direct)}^{ta}\times \frac{z^{i\sim ta}}{l^{j}}$$

(17) $$CE^{i\sim ts}=(CE_{total\;}^{ts}-CE_{direct)}^{ts}\times \frac{z^{i\sim ts}}{l^{j}}$$

### 3.4. Assessment of the Eco-Efficiency of Tourism Sectors of Gansu Province

DEA has distinct advantages in sustainable development assessments and has been a popular tool in recent years for analyzing eco-efficiency [57]. We built a slack-based measure (SBM) model that includes undesirable outputs [58] in order to estimate the annual eco-efficiency of each sector in Gansu’s tourism industry between 1997 and 2016, and using each sector in each year of this period as a decision-making unit, relative efficiency analysis was performed. Each decision-making unit includes three vectors—input, desirable output, and undesired output—and they are denoted as $xx\in R^{p}$, $yy^{g}\in R^{s_{1}}$, and $yy^{b}\in R^{s_{2}}$, respectively. Matrices *W*, *W^g^*, and *W^b^* are defined as follows: $\left[XX\right]={\left[x_{1},\cdots ,x_{q}\right]}^{T}\in R^{p\times q}$, $\left[YY^{g}\right]={\left[yy_{1}^{g},\cdots ,yy_{q}^{g}\right]}^{T}\in R^{s_{1}\times q}$, and $\left[YY^{b}\right]={\left[yy_{1}^{b},\cdots ,yy_{q}^{b}\right]}^{T}\in R^{s_{2}\times t}$, where *XX* > 0, *YY^g^* > 0, and *YY^b^* > 0. The production possibility set *P* is defined as $P=\left\{\left(xx,yy^{g},yy^{b}\right)|xx\geq XX\lambda ,yy^{g}\leq YY^{g}\lambda ,yy^{b}\geq YY^{b}\lambda ,\lambda \geq 0\right\}$. The undesirable-SBM model, which varies with returns to scale, is expressed as follows [59]:

$$EE=min\frac{1-\frac{1}{p}\sum_{d=1}^{p}\frac{s_{i}^{-}}{xx_{i0}}}{1+\frac{1}{s_{1}+s_{2}}\left[\sum_{r=1}^{s_{1}}\frac{s_{r}^{g}}{yy_{r0}^{g}}+\sum_{r=1}^{s_{2}}\frac{s_{r}^{b}}{yy_{r0}^{b}}\right]}$$

$$\mathrm{St}.\;x_{0}=XX\lambda +s^{-}$$

$$yy_{0}^{g}=YY^{g}\lambda -s^{g}$$

$$yy_{0}^{b}=YY^{b}+s^{b}$$

(18) $$\lambda \geq 0,s^{-}\geq 0,s^{g}\geq 0,s^{b}\geq 0$$

where *s* denotes the input and output slack variables and λ is the intensity vector. The objective function, EE, strictly decreases with $s^{-}\in R^{p}$, $s^{g}\in R^{s_{1}}$, and $s^{b}\in R^{s_{2}}$ and 0≤EE≤1.

### 3.5. Input–Output Indicators for Eco-Efficiency Assessments Based on Carbon Emissions

In a traditional economic system, such as in the Cobb–Douglas production function, the input productive factors mainly include labor and capital. As such, in this article, the number of employees, original cost of fixed assets, and operating revenue are taken as the input–output indicators for the economic system, and the direct and total carbon emissions are taken as the undesirable output in order to measure the ecology and climate change impact of tourism sectors (Table 2).

### 3.6. Analysis of Drivers

The Tobit regression model is proposed by Tobin [60]. It belongs to a regression model with limited dependent variables. It can solve the problem of modeling restricted or truncated dependent variables. The Tobit model has been widely used to investigate the influencing factors of eco-efficiency. Because eco-efficiency evaluated by undesirable-SBM always has a value from 0 to 1, it is not suitable to use ordinary least squares (OLS) for coefficient estimation [61]. Therefore, we selected a timeseries Tobit regression model to identify the driving factors of tourism sector eco-efficiency in Gansu. The model expression is as follows [62]:

$$ee_{t}^{*}=\alpha zz_{t}+\varepsilon_{t}$$

(19) $$ee_{t}=\{\begin{array}{c} ee_{t}^{*},\;ee_{t}^{*}\geq 0\;\\ 0,\;ee_{t}^{*}\leq 0 \end{array}\;t=1,\cdots ,\;\;NN\;$$

$$\varepsilon_{t}\sim NN\left(0,\sigma^{2}\right)$$

where *t* denotes the year, *zz_t_* is an independent variable, α is a regression coefficient, and $\varepsilon_{t}$ represents a disturbance term.

For eco-economic theory and existing research, industry scale, capita, structure, and low-carbon technology are the main factors that influence the eco-efficiency [63]. Based on the existing research and the characteristics of the tourism sectors of Gansu, indicators that measure scale effect, structure effect, capital effect, and technological effect were selected for econometric regression analysis, and the drivers of tourism sector eco-efficiency were explored.

Scale effect: In the tourism economic system, according to the theory of returns to scale, with an increase in tourist reception, the production scale continues to expand, and the marginal cost may also decrease [64]. Therefore, the scale effect can improve the eco-efficiency of tourism sectors throughout the economic system. With rapid tourist reception expansion, however, a decline in tourism sectors in Gansu with respect to the marginal production costs, driven by scale effect, may not be able to offset rapidly increasing carbon emissions and eco-environmental pressure. Therefore, further discussion is needed via the regression model. The scale effect is presented by each sector’s total revenue from tourism and the number of tourists served; the indicators in this category include the revenue of star-rated hotels (HTI), revenue of travel agencies (TTI), revenue of scenic spots (STI), number of guests served by star-rated hotels (HTP), number of tourists served by travel agencies (TTP), and number of visitors to scenic spots (STP).

Structure effect: The optimization of industrial structure can reduce the consumption of resources and energy, improve energy utilization efficiency, reduce carbon emissions, and promote the stability and coordination of the ecological economic system [65]. With the tourism industry structure changed, new linkages will be established between tourism sectors, which might affect carbon emission levels and the eco-efficiency of each sector. As such, in this article, the structure effect is mainly measured by the proportion that each sector’s revenue accounts for in the total revenue among the three tourism sectors, including the proportion of the revenue of star-rated hotels (HS), the proportion of travel agency revenue (TS), and the proportion of scenic spot revenue (SS).

Capital effect: As an economic system, the impact of capital input on tourism economic growth is apparent. However, the impact of capital input on the eco-efficiency, or even production efficiency, of tourism is not clear [66]. Whether higher capital input could improve tourism eco-efficiency needs to be further verified. The capital-driven effect is mainly measured using capital input per unit of tourism revenue. This article chooses the original value of fixed capital per unit income in its analysis in order to measure the capital-driven effects of capital investments. The indicators for star-rated hotels, travel agencies, and scenic spots are HRI, TRI, and SRI, respectively.

Technological effect: Low-carbon technology can improve the eco-efficiency of tourism by reducing carbon emissions. The energy efficiency can reflect the carbon efficiency, and it has been a key index to measure low-carbon technology [67]. The technology effect is mainly measured with energy input per unit of tourism revenue in this article. The indicators for star-rated hotels, travel agencies, and scenic spots are as follows: HEI, TEI, and SEI, respectively.

Meanwhile, based on the drivers of eco-efficiency in the industry, this article selects GDP [68], industry structure [65], urbanization [69], civilization [70], open policy [71], and traffic conditions [72] as control variables outside the tourism eco-economic system. The above control variables are represented by per GDP (PGDP), proportion of tertiary industry (THI), proportion of urban population (UR), number of students in colleges and universities (ED), total investment of foreign enterprises (FR), and road mileage (RO), respectively.

The driver analysis examines direct carbon emission eco-efficiency (HDE, TDE, and SDE) and total carbon emission eco-efficiency (THE, TTE, and STE) for each tourism sector. To avoid data issues brought about by variables of different dimensions, and to reduce the heteroscedasticity of variables and enhance data stability, except for ratios and data results less than 1, all other data are taken as their logarithm. The revenue of each sector is adjusted for inflation based on the consumer price index of Gansu Province, with 1997 being the base year. An ADF unit root test was performed for each variable, and differential processing was performed for variables that did not pass the unit root test. Tobit regression was then conducted based on the processed data. The description of variables is motioned above in Table 3.

Based on the above analyses and assumptions, eco-efficiency models under the direct carbon emission scenario and total carbon emission scenario are constructed for star-rated hotels, travel agencies, and scenic spots as follows:

Model 1: Regression model for the eco-efficiency of star-rated hotels:

(20) $$HDE=\alpha_{10}+\alpha_{11}HS+\alpha_{12}HEI+\alpha_{13}lnHTI+\alpha_{14}lnHTP+\alpha_{15}lnHRI\;\;\;\;\;+\alpha_{16}lnPGDP_{\mathrm{control}}+\alpha_{17}THI_{\mathrm{control}}+\alpha_{18}UR_{\mathrm{control}}\;\;\;\;\;\;\;+\alpha_{19}lnED_{\mathrm{control}}+\alpha_{110}lnRO_{\mathrm{control}}+\alpha_{111}lnFR_{\mathrm{control}}+\varepsilon$$

Model 2: Regression model for the eco-efficiency of star-rated hotels with respect to the direct carbon emission scenario:

(21) $$HTE=\alpha_{20}+\alpha_{21}HS+\alpha_{22}HEI+\alpha_{23}lnHTI+\alpha_{24}lnHTP+\alpha_{25}lnHRI\;\;\;\;\;+\alpha_{26}lnPGDP_{\mathrm{control}}+\alpha_{27}THI_{\mathrm{control}}+\alpha_{28}UR_{\mathrm{control}}\;\;\;\;\;\;\;+\alpha_{29}lnED_{\mathrm{control}}+\alpha_{210}lnRO_{\mathrm{control}}+\alpha_{211}lnFR_{\mathrm{control}}+\varepsilon$$

Model 3: Regression model for the eco-efficiency of travel agencies:

(22) $$TDE=\alpha_{30}+\alpha_{31}TS+\alpha_{32}TEI+\alpha_{33}lnTTI+\alpha_{34}lnTTP+\alpha_{35}TRI\;\;\;\;\;+\alpha_{36}lnPGDP_{\mathrm{control}}+\alpha_{37}THI_{\mathrm{control}}+\alpha_{38}UR_{\mathrm{control}}\;\;\;\;\;\;\;+\alpha_{39}lnED_{\mathrm{control}}+\alpha_{310}lnRO_{\mathrm{control}}+\alpha_{311}lnFR_{\mathrm{control}}+\varepsilon$$

Model 4: Regression model for the eco-efficiency of travel agencies with respect to the direct carbon emission scenario:

(23) $$TTE=\alpha_{40}+\alpha_{41}TS+\alpha_{42}TEI+\alpha_{43}lnTTI+\alpha_{44}lnTTP+\alpha_{45}TRI\;\;\;\;\;+\alpha_{46}lnPGDP_{\mathrm{control}}+\alpha_{47}THI_{\mathrm{control}}+\alpha_{48}UR_{\mathrm{control}}\;\;\;\;\;\;\;+\alpha_{49}lnED_{\mathrm{control}}+\alpha_{410}lnRO_{\mathrm{control}}+\alpha_{411}lnFR_{\mathrm{control}}+\varepsilon$$

Model 5: Regression model for the eco-efficiency of tourist agencies:

(24) $$SDE=\alpha_{50}+\alpha_{51}SS+\alpha_{52}SEI+\alpha_{53}lnSTI+\alpha_{54}lnSTP+\alpha_{55}SRI\;\;\;\;\;+\alpha_{56}lnPGDP_{\mathrm{control}}+\alpha_{57}THI_{\mathrm{control}}+\alpha_{58}UR_{\mathrm{control}}\;\;\;\;\;\;\;+\alpha_{59}lnED_{\mathrm{control}}+\alpha_{510}lnRO_{\mathrm{control}}+\alpha_{511}lnFR_{\mathrm{control}}+\varepsilon$$

Model 6: Regression model for the eco-efficiency of scenic spots with respect to the direct carbon emission scenario:

(25) $$STE=\alpha_{60}+\alpha_{61}SS+\alpha_{62}SEI+\alpha_{63}lnSTI+\alpha_{64}lnSTP+\alpha_{65}SRI\;\;\;\;\;+\alpha_{66}lnPGDP_{\mathrm{control}}+\alpha_{67}THI_{\mathrm{control}}+\alpha_{68}UR_{\mathrm{control}}\;\;\;\;\;\;\;+\alpha_{69}lnED_{\mathrm{control}}+\alpha_{610}lnRO_{\mathrm{control}}+\alpha_{611}lnFR_{\mathrm{control}}+\varepsilon$$

### 3.7. Data Sources

Data on the number of employees, original cost of fixed assets, operating revenue, and number of tourists receipted were obtained from the Yearbook of China Tourism Statistics. The input–output data for calculating total carbon emissions were obtained from input–output tables in the Statistical Yearbook of Gansu Province for the years 1997, 2002, 2007, and 2012. Data on the value added by industry for each year were obtained from the Statistical Yearbook of Gansu Province for the years 1997 to 2017. Per GDP, proportion of tertiary industry, proportion of urban population, number of students in colleges and universities, total investment of foreign enterprises, and road mileage were obtained from the Statistical Yearbook of Gansu Province from 1998 to 2017. Fixed assets, operating revenue, per GDP, and total investment of foreign enterprises were all adjusted for inflation based on the consumer price index of Gansu Province, with 1997 being the base year.

## 4. Results and Discussion

### 4.1. The Carbon Emissions of Tourism Sectors’ in Gansu Province

**Figure 3:**
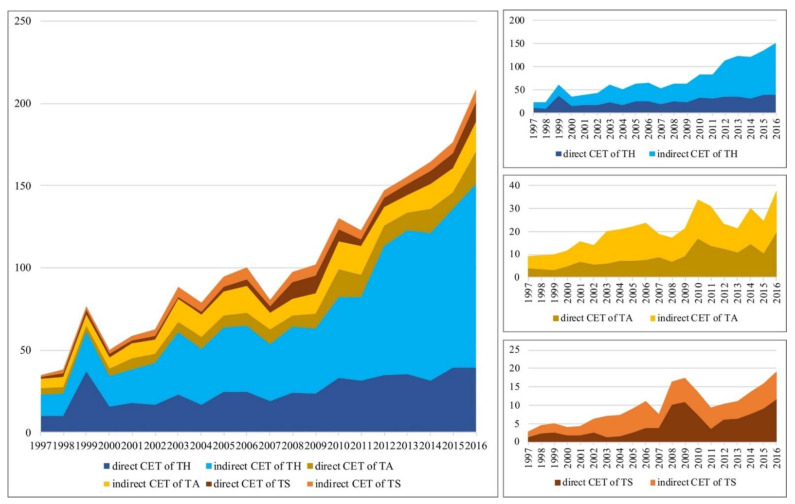
Carbon emissions of tourism sectors in Gansu Province during the 1997–2016 period.

The total carbon emissions of the three tourism sectors in Gansu increased from 50.6 kilotons in 1997 to 229 kilotons in 2016, with an average growth of 18.25%, a little higher than that of the 16.01% in China’s tourism industry, as evaluated by Zha [73]. The indirect part of carbon emissions increased from 55.7 kilotons in 1997 to 173.3 kilotons in 2016. Indirect carbon emissions account for 65.9% of total carbon emissions, an increase of 1.93 times more due to direct carbon emissions from tourism industry in 2016. The average ratio of indirect emissions within total carbon emissions has similarities to data in related research. This ratio was 57.5%, 64%, and 52% in China [74], New Zealand [75] and Australia [76], respectively. The growth of indirect emissions was faster than that of direct emissions. The carbon emissions of tourism hotels in Gansu were more than travel agencies and scenic spots; this result is consist with the situation in China [74] and globally [7]. By 2016, the amount of indirect carbon emissions from tourism hotels was 6.13 times and 14.54 times those of travel agencies and scenic spots, respectively. Scenic spots saw relatively fast growth in direct emissions, slightly higher than those of tourism hotels and travel agencies (Figure 3).

Indirect carbon emissions from tourism hotels grew rapidly during the 1997–2016 period. The evolution of the composition of carbon emissions indicates that a growing number of emissions from tourism hotels were indirect emissions caused by related industries, and that incremental emissions mainly stemmed from intermediate production steps (Figure 3, top right). From 1997 to 2016, the direct carbon emissions of tourism hotels in Gansu increased from 10,200 tons to 39,500 tons, representing an annual growth of 7.4%, which was lower than the average rate of the economic growth (operating revenue), similar to that of Chinese accommodation and food in general with 7.5% [74]. From 1997 to 2016, indirect carbon emissions increased from 13,100 tons to 111,600 tons, representing a growth of 8.5 times across the period, with an annual increase of 11.3%, which was higher than that of China’s general increase of 7.5% [74]. The proportion of indirect carbon emissions from tourism hotels in the total indirect carbon emissions increased from 57.19% in 1997 to 73.84% in 2016. Although carbon emissions from tourism hotels with respect to providing final products did not increase substantially during the study period, emissions from intermediate inputs and outputs increased significantly. In 1997, Gansu Province had 38 tourism hotels, increasing to 299 as of 2016, six times more than it had in 1997. The scale growth was fast during this period. However, the development of higher tourism hotels was slow. As of 2016, Gansu only had three five-star hotels, and they were all in the provincial capital city of Lanzhou. The lagging development of high-standard hotels may be a main factor leading to high carbon emissions from tourism hotels in Gansu. The high indirect carbon emissions indicate that industries related to hotels had high carbon emissions, and that this phenomenon was related to low-carbon and energy efficiency in Gansu Province being backward [77]. In summary, there is considerable room for hotels and related industries in Gansu to reduce carbon emissions, and there is also a need to comprehensively regulate related industries.

Both direct and indirect carbon emissions from travel agencies in Gansu Province were high and showed a fluctuating pattern. Direct carbon emissions increased from 4000 tons in 1997 to 19,900 tons in 2016, representing an annual growth of 8.8%, which was higher than the annual growth rate of direct carbon emissions from tourism hotels. Indirect carbon emissions increased from 5100 tons in 1997 to 18,100 tons in 2016, an annual increase of 6.5%; this growth was lower than that of the direct emissions of travel agencies and far below the annual growth in the indirect emissions of tourism hotels. The contribution of indirect emissions to the total emissions of travel agencies declined annually. The impact of intermediate production steps on carbon emissions decreased annually, and carbon emissions were increasingly attributed to the production of final products (Figure 3, middle right). Travel agencies in Gansu lagged in terms of developing online services, and they focused on employing traditional offline sale channels to promote local tourist routes. This led to considerably more tourism activities related to traveling to other provinces or countries than those involving coming to Gansu. Therefore, Gansu’s travel agencies did not have a high demand for other related industries locally, and they did not require significant local input in their operations. As a result, the carbon emissions of travel agencies via intermediate production steps were not high.

The total carbon emissions of scenic spots were lower than those of tourism hotels and travel agencies, but the growth rate was far above those for the other two sectors, especially with respect to direct carbon emissions. Direct carbon emissions from scenic spots increased from 1200 tons in 1997 to 11,700 tons in 2016, with an annual growth rate of 12.7%. The total carbon emissions of scenic spots increased from 2800 tons in 1997 to 19,400 tons in 2016, representing an annual growth rate of 10.7%. The direct and indirect carbon emissions were both higher than sightseeing carbon emissions in China from 2002 to 2010, with 3.8% and 3%, respectively [74]. In terms of the evolutionary trajectory, carbon emissions from scenic spots peaked in 2008 and 2009 and increased rapidly again in recent years. This was correlated with a significant increase in the number of visitors to Gansu’s scenic spots as a result of the development of major scenic spots and high-speed train services; it is also an indication that there was a stronger correlation between the level of carbon emissions and the scale of the tourism industry in the development of scenic spots than in the development of tourism hotels or travel agencies (Figure 3, bottom right). Gansu has few large-scale, high-quality, national scenic spots (e.g., 5A attractions). Coordinated development between scenic spots and other industries is also low. As a result, scenic spots have low indirect carbon emissions. The continuous, rapid increase in direct emissions indicates that, currently, the final products and services of Gansu’s scenic spots are provided to visitors without low-carbon technology. In comparison to vacation-oriented tourist destinations that have a strong supply chain effect, for example, Jamaica [8], sightseeing is the major tourist activity in Gansu, and few visitors stay overnight. With the rapid increase in the number of visitors, scenic spots in Gansu had not developed an economic influence on the surrounding regions, and their connections with other industries are weak; this explains why these scenic spots had low indirect emissions but a rapid growth in direct emissions.

### 4.2. The Sources of Indirect Carbon Emissions from the Supply Chain of Tourism Sectors

**Figure 4:**
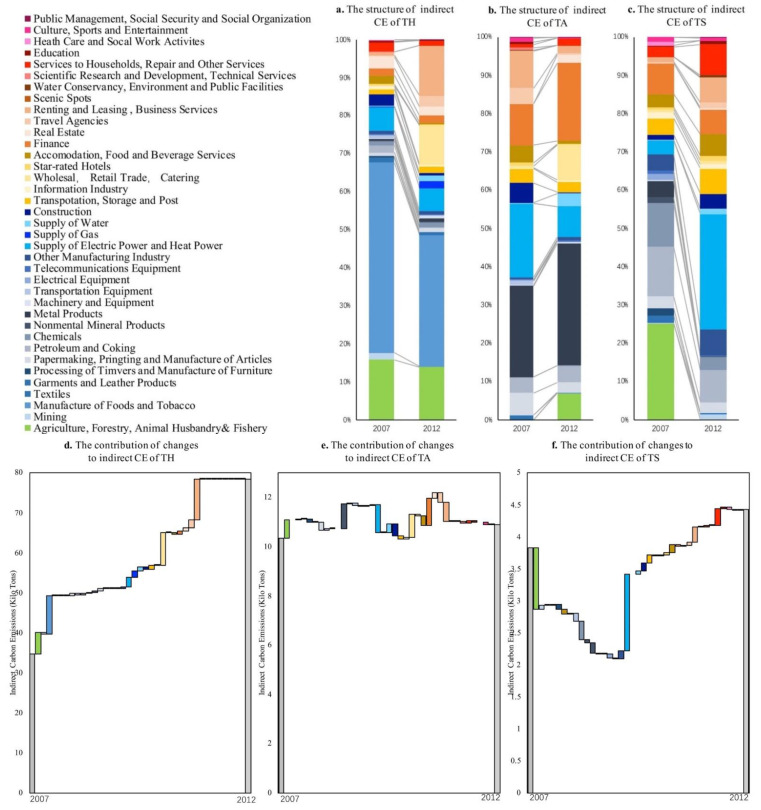
The main sources of indirect carbon emissions from tourism sectors in Gansu Province.

Via Equations (15)–(17), the sources of indirect carbon emissions can be obtained. The manufacturing of food and tobacco was the main source of the indirect carbon emissions of tourism hotels in Gansu; furthermore, the contribution of renting, leasing, and business services to the indirect carbon emissions of tourism hotels has increased more obviously in recent years, and they have become the main contribution sectors to the increase in indirect carbon emissions from tourism hotels in Gansu. In 2007, the main source of indirect carbon emissions from tourism hotels in Gansu was from the manufacturing of food and tobacco, accounting for 50%. In 2012, the main source of indirect carbon emissions from tourism hotels was the manufacturing of foods and tobacco, accounting for 34.5% (Figure 4a). From 2007 to 2012, the contribution of indirect carbon emissions from tourism hotels in Gansu mainly came from renting, leasing, and business services; the manufacturing of foods and tobacco; wholesale; retail trade; catering; agriculture; forestry; animal husbandry and fishery; and supplying electric and heat power, contributing increments of 10.1, 9.8, 8.1, 5.4, and 2.5 kilotons, respectively (Figure 4d).

Metal products were the main source of the indirect carbon emissions of travel agencies in Gansu. The contribution of finance has increased in recent years, which became the main contribution sector with respect to the increase in the carbon emissions of travel agencies in Gansu. In 2007, the main source of indirect carbon emissions from travel agencies in Gansu was metal products, accounting for 23.8%. In 2012, the main source of indirect carbon emissions from Gansu travel agencies was metal products, accounting for 31.7% (Figure 4b). From 2007 to 2012, the contribution to the increase in carbon emissions from travel agencies in Gansu mainly came from finance, metal products, wholesale, retail trade, catering, agriculture, forestry, animal husbandry and fishery, and the supplying of water, contributing increments of 1.1 kilotons, 1 kilotons, 0.9 kilotons, 0.8 kilotons, and 0.3 kilotons of indirect carbon emission, respectively (Figure 4e).

Agriculture, forestry, animal husbandry and fishery, and supplying electric and heat power were the main sources of indirect carbon emissions from scenic areas in Gansu, and carbon emissions from supplying electric and heat power have increased obviously in recent years, and have become the main contribution sector with respect to the increase in carbon emissions from scenic areas in Gansu. In 2007, the main sources of indirect carbon emissions in scenic spots in Gansu were agriculture, forestry, and animal husbandry and fishery, accounting for 25%. In 2012, the main source of indirect carbon emissions from scenic spots in Gansu was from supplying electric and heat power, accounting for 30% (Figure 4c). For the years 2007–2012, the indirect carbon emissions increased in Gansu scenic spots mainly due to supplying electric and heat power; household services, repair and other services; renting, leasing, and business services; and other manufacturing industries, transportation, storage, and post, contributing increments of 1.2, 0.3, 0.2, 0.1, and 0.1 kilotons, respectively (Figure 4f).

### 4.3. Analysis of the Eco-Efficiency of Tourism Sectors in Gansu

**Figure 5:**
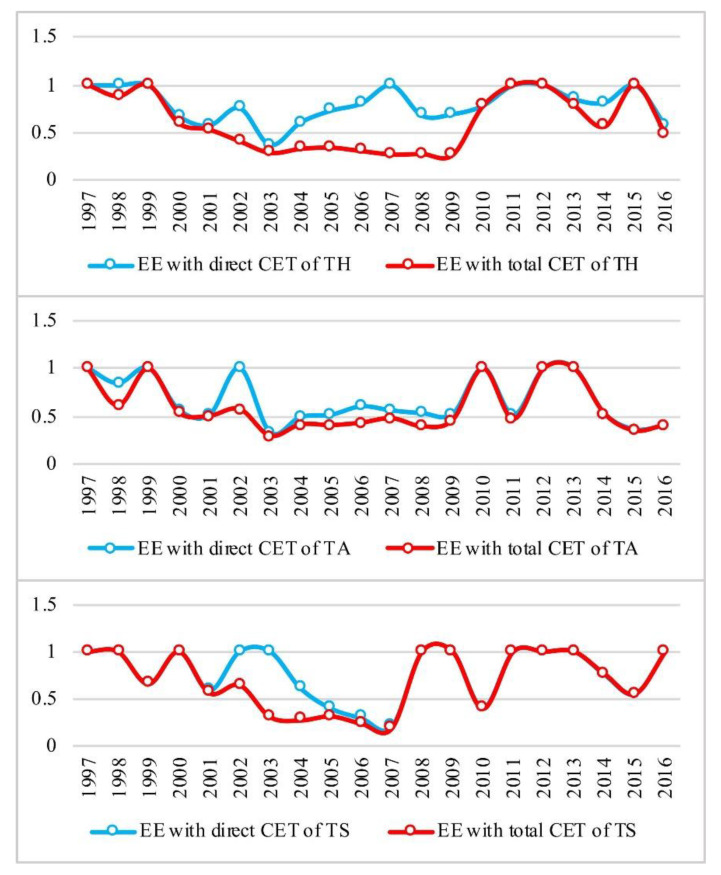
Eco-efficiency of tourism sectors in Gansu Province during the 1997–2016 period.

During the study period, with the increase in revenue, the eco-efficiency of tourism sectors in Gansu demonstrated a U-shaped development pattern in which eco-efficiency first decreased and then increased. This pattern coincides with the environmental Kuznets curve theory [18]. Some scholars have also found similar results between tourism development and environmental impact [18,78], or in the eco-efficiency of urban [79] and regional [80] development. Considering both the direct carbon emission and total emission scenarios, the ranking of the eco-efficiency of the three sectors is as follows: tourism hotels > travel agencies > scenic spots. The eco-efficiency of these tourism sectors entered an evident trough period from 2002 to 2006. Between 2007 and 2011, the eco-efficiency of tourism hotels, travel agencies, and scenic spots started to recover. After 2012, as a result of the latest round of the province’s policy stimulus and high-level infrastructure development, including the construction of a high-speed rail, the eco-efficiency of tourism hotels, travel agencies, and scenic spots returned to a high level (Figure 5).

The eco-efficiency of tourism hotels with respect to total carbon emissions demonstrated a distinct U-shaped pattern. In most years during the study period, eco-efficiency with respect to the direct emission scenario was higher than that of the total emission scenario. In 2003 and 2008, eco-efficiency in both scenarios was at the bottom, indicating low eco-efficiency. During these periods, tourism hotels did not experience an evident increase in either direct or total carbon emissions, indicating that carbon emissions did were not constrained, which did not improve the eco-efficiency of tourism hotels. However, in the early stage of tourism development in Gansu, during which tourism hotels had a limited number of guests and, therefore, low economic efficiency, eco-efficiency was low. As of 2012, the eco-efficiency of tourism hotels with respect to both the direct carbon emission and indirect emission scenarios peaked. At the same time, both direct and total carbon emissions increased somewhat but did not have a major impact on eco-efficiency. As such, the economic contributions of tourism hotels may have compensated for the increase in carbon emissions.

The eco-efficiency of travel agencies with respect to total carbon emissions displayed a U-shaped development pattern and was at the trough of the U shape during the 2003–2009 period. By 2009, eco-efficiency with respect to the direct and total emission scenarios demonstrated recovering yet fluctuating trends; the two types of eco-efficiency, however, fell into a trough again in both 2011 and 2015. Although the eco-efficiency of travel agencies also peaked in 2012, compared to tourism hotels, travel agencies exhibited more eco-efficiency fluctuations after 2012. This means that the interactive relationship between carbon emissions from travel agencies and economic development is less stable and more complex. The evolutionary trajectory of eco-efficiency with respect to both the direct and total emission scenarios was relatively consistent, and the increase in total carbon emissions did not lead to reduced eco-efficiency for travel agencies. This indicates that travel agencies operated with higher efficiency when they considered their connections to other industries; that is, with an open strategy, travel agencies may better balance economic growth and carbon emission reduction.

The evolutionary trajectory of scenic spot eco-efficiency with respect to direct and total carbon emissions was consistent and mostly increased after 2007. Between 2002 and 2006, the eco-efficiency of scenic spots under the direct emission scenario was significantly higher than that of the total emission scenario. In comparison with those of tourism hotels and travel agencies, the eco-efficiency of scenic spots was even lower. The development of scenic spots in Gansu lags behind the national level. After 2000, scenic spot development in Gansu gained momentum, because scenic spots that were previously public agencies underwent a systematic transformation, and the reform constituted a rare opportunity to spur economic growth in scenic spots. Moreover, since 2012, due to construction of the high-speed railway, scenic spots in Gansu experienced a growth spurt, especially in the Hexi Corridor area. The abrupt increase in the number of tourists created more economic benefit but also led to increased carbon emissions. As a result, the eco-efficiency of scenic spots with respect to both direct and indirect carbon emissions declined. Moreover, after 2012, the continuous growth of direct carbon emissions instead brought a higher eco-efficiency, which indicates that the provision of scenic spots as a product of the entire tourism industry supply chain is conducive to improvements in eco-efficiency.

### 4.4. Analysis of the Drivers of Tourism Sector Eco-Efficiency in Gansu

The regression results indicate that both the structure effect and energy technology effect had a significantly positive effect on the eco-efficiency of tourism hotels with respect to total carbon emissions and direct carbon emissions, respectively. An increase of 1% in the revenue of tourism hotels increased their eco-efficiency with respect to direct and total carbon emissions by 1.76 times and 1.84 times, respectively. A reduction of 1% in tourists of tourism hotels increased the eco-efficiency of tourism hotels with respect to direct and total carbon emissions by 1.2 times and 1.36 times, respectively. An increase of 1% in the unit revenue of investment increased the eco-efficiency of tourism hotels with respect to direct and total carbon emissions by 1.68 times and 1.38 times, respectively. A 1% reduction of the unit revenue of energy consumption increased the eco-efficiency of tourism hotels with respect to direct carbon emissions by 3.36. An increase of 1% in the proportion of the revenue of tourism hotels in the total revenue of the three sectors increased the eco-efficiency of tourism hotels with respect to total carbon emissions by 1.52 times (Table 4). Tourism revenue played a more positive role in the eco-efficiency of tourism hotels with respect to total carbon emissions than it did with respect to direct carbon emissions. However, the number of tourist receptions in the scale effect had a significant negative correlation with the eco-efficiency of tourism hotels. This indicates that, because of the relatively lagging development of tourism hotels in Gansu Province, the tourist consumption on tourism hotels was lower. The capital effect was the main driver for the improvement of the eco-efficiency of tourism hotels with respect to both emission scenarios. This is related to the characteristics of high capital investment in tourism hotels [50], and it is also related to the rapid development and large-scale construction of tourism hotels in Gansu Province during the research period.

The structure effect and energy technology effect influenced the eco-efficiency of travel agencies with respect to both direct and total carbon emissions. An increase of 1% in the proportion of the revenue of travel agencies in the total revenue of the three sectors decreased the eco-efficiency of travel agencies with respect to direct carbon emissions and total carbon emissions by 3.16 times and 2.29 times. A 1% reduction in the unit revenue of energy consumption increased the eco-efficiency of travel agencies with respect to direct carbon emissions and total carbon emissions by 5.29 times and 3.33 times (Table 4). The structure of Gansu’s tourism industry, which has a significant negative impact on the eco-efficiency of travel agencies, is key to improving the eco-efficiency of this sector. The energy technology effect has a more significant impact on direct carbon emissions, and the elasticity coefficient is also greater; that is, changes in travel agency eco-efficiency with respect to direct carbon emissions are more sensitive to changes in energy technology. This result also indicates that the travel agency sector in Gansu is not well developed—the operation mechanism and processes are still backward, especially for tourists coming to Gansu. A model that focuses on traveling abroad does not make a significant contribution to the development of local tourism. As such, it is imperative to adjust the internal structure of travel agencies and enhance their modernization in order to improve reception capacity and quality. Furthermore, indirect, coordinated interindustry operations can be adopted to increase the influence of tourism hotels and scenic spots on the tourism industry. The energy technology effect had a significant positive impact on the eco-efficiency of travel agencies in Gansu. The main reason is that the distribution of tourism resources in Gansu is relatively scattered, and the mature, international tourism routes along the Silk Road (Tianshui–Lanzhou–Zhangye–Jiayuguan–Dunhuang) have long distances, contributing to increased energy consumption and carbon emissions.

In regard to the structure effect, the scale effect and energy technology effect influence the eco-efficiency of scenic spots with respect to both direct and total carbon emissions. The capital effect has a relatively significant effect on the eco-efficiency of scenic spots with respect to total carbon emissions. An increase of 1% in the proportion of the revenue of scenic spots in the total revenue of the three sectors decreased the eco-efficiency of scenic spots with respect to direct carbon emissions and total carbon emissions by 16.04 times and 2.29 times, respectively. An increase of 1% in the revenue of scenic spots decreased the eco-efficiency of scenic spots with respect to direct and total carbon emissions by 7.71 times and 1.32 times, respectively. An increase of 1% in the tourists of scenic spots increased the eco-efficiency of scenic spots with respect to direct and total carbon emissions by 3.7 times and 0.79%, respectively. A 1% reduction of in the unit revenue of energy consumption increased the eco-efficiency of scenic spots with respect to direct and total carbon emissions by 81.44 times and 9.36 times, respectively. An increase of 1% in the unit revenue of investment increased the eco-efficiency of scenic spots with respect to direct carbon emissions by 19 times (Table 4). The effect of energy technology with respect to direct carbon emissions was particularly prominent. The effect of the scale effect on scenic spots was the opposite of its effect on tourism hotels, which shows that the current per capita consumption level of scenic spots needs to reduced, since the increase in reception has not improved the eco-efficiency of Gansu scenic spots. This situation should be fully considered due to being in the left half of the environmental Kuznets curve. Therefore, attention should be paid to the development and use of green and low-carbon technologies for tourism products and services in scenic spots given the resilient energy technology effect in order to build a low-carbon and high-quality scenic spot using efficient and guided investment.

## 5. Discussion

According to Kuznets’ environmental theory, the preliminary results of tourism sector low-carbon development have been achieved in Gansu, and the aim of carbon emissions peak is expected to be achieved soon. The growth of indirect emissions was faster than that of direct emissions, which coincided with Gansu’s tourism development at that phase: The industry was evolving and maturing, i.e., transitioning from supplying a single product to providing comprehensive and diversified tourism services [81]. Indirect carbon emissions were 1.93 times that of direct carbon emissions with respect to Gansu tourism, lower than that of the global tourism industry, which is four times larger [7]. Compared with the other industries, such as agriculture and manufacturing, tourism has, relatively, the lowest direct carbon emissions [74]. Moreover, tourism sectors have gained a high eco-efficiency with a faster increase in economic growth compared to carbon emissionsas a result of regional policy stimulus, high-level infrastructure development, higher management efficiency, fairer allocation of resources [82]: specific like the construction of high-speed rails, a more efficient and reasonable development of scenic spots. Our results show that the preliminary fruits of each sector’s low-carbon development have been achieved, and the aim of carbon emissions peak is expected to soon be achieved in underdeveloped areas in northwest China. In the future, we should adhere to the concept of green development, adhere to promoting the decoupling of tourism carbon emissions from economic growth, scientifically evaluate the development status of tourism sectors, promote the inflection point of the Kuznets curve of carbon emissions with respect to tourism sectors, and strengthen top-level designs.

Tourism hotels being the main contributor of indirect carbon emissions from the supply chain confirms other scholars’ research on the increasing food consumption of tourists [83]. The structure of indirect carbon emissions with respect to the tourism sector in Gansu is mostly consistent with the results of Lenzen’s study on low-income countries [7]. Based on the existing research, structure changes were an important factor in offsetting indirect carbon emissions in China during 1997–2012 [84]. Therefore, in the new era, China has made efforts to change its economic development mode, targeting high-quality economic growth, i.e., growth driven by higher value added and lower resource intensive inputs [84]. As an important emissions contributor in China, Gansu is taking numerous measures to save energy and reduce carbon emissions. Therefore, industry structural changes, both in tourism and other whole industries, are expected to continue to decrease indirect carbon emissions from the supply chain in the future. The tourism sector should pay attention to coordinated development in the supply chain with respect to indirect carbon emission sources in order to promote carbon emission efficiency.

The drivers of eco-efficiency in the tourism sector are consist with Luo’s analysis of the drivers of carbon emissions in China’s tourism industry [67]. Moreover, the world is shifting to the use of renewable energy sources [85], and China has taken many effective measures to improve energy efficiency [86]. The structure effect has certain positive effects on tourism hotels and scenic spots, but it has certain negative impacts on travel agencies. Therefore, with the increase in tourism revenue and the share of tourism hotels and scenic spots, and the reduction in the share of travel agency income in all three industries, the structure effect can improve the eco-efficiency of the three sectors. Due to the different effects that the scale effect has on the three sectors, tourism hotels should be able to improve eco-efficiency by improving their per capita income levels, while travel agencies and scenic spots should reduce their per capita consumption levels and expand the reception capacity in order to improve eco-efficiency. Therefore, the green development of tourism sectors in Gansu Province in the future should be driven by improvements in product and service quality, encourage tourism enterprises to provide low-carbon tourism products from the supply side in order to guide green tourism consumption, and avoid blindly expanding the market scale.

## 6. Conclusions

Taking tourism hotels, travel agencies, and scenic spots in Gansu, China, as study objects, the direct and indirect carbon emissions of the three sectors were measured through EEIO, the eco-efficiency of the three sectors with respect to the direct and total emission scenarios was calculated using the DEA model, and the factors that drive the eco-efficiency of each sector were analyzed. The major conclusions obtained are as follows:

The carbon emissions of Gansu’s three tourism sectors continuously increased, especially indirect emissions. The evolution of the eco-efficiency of the three tourism sectors all demonstrated a U-shaped pattern.

Food and tobacco production was the main contributor of indirect carbon emissions from the supply chain of tourism hotels, which contributed the most carbon emissions in the tourism sector, followed by unprocessed food (listed under agriculture, forestry, animal husbandry and fishery).

Energy technology is the key driver in improving the eco-efficiency of the tourism sectors in Gansu. Specifically, the structure effect and energy technology effect had a significantly positive effect on the eco-efficiency of tourism hotels. The structure effect and energy technology effect influenced the eco-efficiency of travel agencies The structure effect, scale effect, and energy technology effect influence the eco-efficiency of scenic spots with respect to both direct and total carbon emissions.

This research constructs a comprehensive research framework regarding tourism sector carbon emissions and the eco-efficiency in order to evaluate carbon emissions and their sources in the tourism sectors with respect to a supply chain with intermediate input sectors, finding a path to accurately judge tourism sector carbon emissions. This research evaluated eco-efficiency with respect to both the direct and total carbon emission scenarios, applying a multiple input, multiple output model to explore the comprehensive effects and driving factors of the tourism sector on eco-economy and to provide a widely used decision-making analysis tool for tourism sectors facing the pressures of economic recovery in the post-COVID-19 era and global climate change.

Limitations of this study: Gansu’s tourism industry is still dominated by mass tourism, and a large part of the carbon emissions from the transportation sector can be reflected through travel agencies, as well as the input–output relationship between scenic spots and hotels. Therefore, this paper does not conduct a separate analysis on traffic carbon emissions, and future research will be required via field work or by taking traffic carbon emissions as a special topic.

Further research: This study takes the whole territory of Gansu as an example. In fact, there are great differences between natural environmental conditions and tourism resource endowments among the 14 cities in Gansu, and there might be spatial differences in tourism carbon emissions and eco-efficiency. Future research will focus on summarizing the spatial differentiation law of tourism eco-efficiency in Gansu, using spatial econometric analysis in order to achieve spatial and precise policy formulation. Moreover, the analysis of direct and indirect carbon emissions and the eco-efficiency of tourism sector at a larger spatial scale will be the focus of future research. For example, the study of spatial differentiation in Mainland China and various provinces will be the main direction of future research.

## Figures and Tables

**Table 1:** Input–output table.

F		Intermediate Use						
	Industrial Sector	S1	…	S*j*	…	Sn	Final Use	Total Output
Intermediate input	IS1	$z^{11}$	…	$z^{1j}$	…	$z^{1n}$	$f^{1}$	$x^{1}$
	⋮	⋮		⋮		⋮	…	…
	ISi	$z^{i1}$	…	$z^{ij}$	…	$z^{in}$	$f^{i}$	$x^{i}$
	⋮	⋮		⋮		⋮	…	…
	ISn	$z^{n1}$	…	$z^{nj}$	…	$z^{nn}$	$f^{n}$	$x^{n}$
Value added		$l^{1}$	…	$l^{j}$	…	$l^{n}$		
Total input		$x\prime^{1}$	…	$x\prime^{j}$	…	$x\prime^{n}$		

Note: The part of table in yellow denotes intermediate inputs and outputs, the part of table in bule denotes final use, the part of table in green denotes value added, and the column in red border denote supply chain.

**Table 2:** Input–output indicators for assessing the eco-efficiency of tourism sectors in Gansu Province based on carbon emissions.

	Indicator	Data Source	Unit
Input	Number of employees	Yearbook of China Tourism Statistics	Count
	Original cost of fixed assets	Yearbook of China Tourism Statistics	10,000 Yuan
Output	Operating revenue	Yearbook of China Tourism Statistics	10,000 Yuan
Undesirable output	Direct carbon emissions/Total carbon emissions	Calculation	10,000 tons

**Table 3:** Description of variables for the analysis of the drivers of tourism sector eco-efficiency in Gansu Province.

Variable	Mean	Standard Deviation	Minimum	Maximum
HDE	0.7936	0.1882	0.3619	1.0000
HTE	0.6014	0.2972	0.2581	1.0000
HS	0.5755	0.0990	0.3243	0.8449
HEI	0.3139	0.0561	0.2464	0.4089
lnHTI	11.5222	0.5330	10.4293	12.1225
lnHTP	9.8961	0.5825	8.7744	10.4335
lnHRI	1.1084	0.3276	0.0000	1.5056
TDE	0.6592	0.2500	0.3227	1.0000
TTE	0.5870	0.2556	0.2835	1.0000
TS	0.2691	0.0631	0.0873	0.3545
TEI	0.2310	0.0419	0.1704	0.3016
lnTTI	10.7397	0.5922	9.5321	11.5509
lnTTP	13.3644	0.5396	12.1093	14.1544
TRI	0.9485	1.2501	0.0680	6.0133
SDE	0.7771	0.2777	0.2175	1.0000
STE	0.6993	0.3128	0.1977	1.0000
SS	0.1554	0.0981	0.0536	0.4709
SEI	0.2310	0.0419	0.1704	0.3016
lnSTI	10.0833	0.9741	8.6770	12.3838
lnSTP	16.3363	1.2287	14.6281	18.3264
SRI	3.2302	2.0131	0.5775	8.1709
lnPGDP	9.0238	0.6053	8.0706	9.8162
THI	0.4075	0.0417	0.3347	0.5141
UR	0.3177	0.0789	0.1839	0.4467
lnED	3.0698	0.7644	1.6233	3.8225
lnRO	1.9817	0.5917	1.2698	2.6603
lnFR	8.1080	0.7943	5.4972	8.9434

**Table 4:** Tobit regression results for factors that drive the tourism eco-efficiency of tourism hotels, travel agencies, and scenic spots in Gansu Province.

	Model 1		Model 2		Model 3		Model 4		Model 5		Model 6	
	Coef.	t	Coef.	t	Coef.	t	Coef.	t	Coef.	t	Coef.	t
HS	0.8167	1.46	1.5175	2.2 *								
D1.lnHTI	1.7599	3.69 ***	1.8436	2.92 **								
D1.lnHTP	−1.2001	−2.77 **	−1.3645	−2.27 *								
D1.HEI	−3.3593	−4.48 ***	−1.5378	−1.67								
D1.lnHRI	1.6817	3.44 **	1.3837	2.43 **								
TS					−3.1697	−2.21 *	−2.2856	−2.7 **				
D1.lnTTI					−0.2767	-1.3	−0.2821	−1.47				
lnTTP					0.7395	1.43	0.7499	1.78				
TEI					−5.2951	−2.62 **	−3.3253	−2.12 *				
TRI					−0.0118	−0.25	−0.0050	−0.13				
SS									16.0436	3.26 **	2.9920	2.39 **
D1.lnSTI									−7.7122	−3.49 **	−1.3180	−2.62 **
D1.lnSTP									3.7003	3.41 **	0.7931	2.23 *
SEI									−81.4394	−3.34 **	−9.3551	−2.45 **
SRI									0.4058	−4.65 ***	−0.0834	−1.72
D2.lnpgdp	−1.2876	−1.65	0.5526	0.58	2.2572	1.2	0.9559	0.75	18.9987	3.22 **	−0.6318	−0.42
D1.thirdi	−2.5387	−0.77	−0.7067	−0.18	−7.1938	−1.65	−8.7556	−2.89 **	40.0462	3 **	−2.4897	−0.43
D1.urban	15.1017	1.68	15.4329	1.04	−3.4939	−0.24	5.2261	0.43	−115.4852	−2.57 **	14.4444	0.82
lnedu	0.0667	0.49	−0.6814	−3.6 ***	−1.1568	−2.63 **	−1.0686	−3.49 **	−9.7988	−3.17 **	−1.3673	−2.26 *
D1.lnroad	0.1216	0.7	0.1952	0.85	−0.3837	−1.38	−0.2979	−1.18	1.8133	3.5 **	0.4618	1.14
lnfr	0.3104	1.83	1.1500	5.47 ***	0.7369	0.98	0.8118	1.52	8.1213	3.06 **	1.5389	2.36 *
_cons	−2.7007	−1.94 *	−7.8254	−4.84 ***	−9.4326	−2.44**	−11.3842	−3.42 **	−14.0173	−2.25*	−5.7348	−1.96 *
Log likelihood	7.8004		6.0796		4.7628		7.3352		3.5624		−1.8279	

Note: *, **, and *** denote significance at the 0.1, 0.05, and 0.01 levels, respectively. D1. and D2. denote first order difference and second difference, respectively.

## Data Availability

The data presented in this study are available in Yearbook of China Tourism Statistics, and Statistical Yearbook of Gansu Province, it can be found at the website: https://navi.cnki.net/knavi/yearbooks/index.

## Nomenclature

Acronyms			
DEA	data envelopment analysis	*XX*	the input matrix of eco-efficiency
IOA	input–output analysis	*YY*	the output matrix of eco-efficiency
SBM	slacks-based measure of efficiency	*NN*	the years of eco-efficiency analysis
EE	eco-efficiency	*z*	the intermediate input/use
EC	the consumption of fuel	*f*	the final use
TR	the total revenue of tourist sector	*l*	the added value
CE	carbon emissions	*x*	the total output
HTI	the revenue of star-rated hotels	*x’*	the total input
TTI	the revenue of travel agencies	*η*	the energy consumption coefficient
STI	the revenue of scenic spots	*δ*	the convert coefficient to standard coal
TTP	number of tourists served by travel agencies	*μ*	the carbon emissions coefficient
STP	number of visitors to scenic spots	*xx*	the input of eco-efficiency
HS	the proportion of star-rated hotels’ revenue	*yy*	the output of eco-efficiency
SS	the proportion of scenic spots’ revenue	*s*	the slack variable
TS	the proportion of travel agencies’ revenue	*ee*	eco-efficiency
HRI	the capital input per unit of star-rated hotels revenue	*zz*	the influencing indicators
TRI	the capital input per unit of travel agencies’ revenue	*ε*	disturbance term
SRI	the capital input per unit of scenic spots’ revenue	*α*	regression coefficient of the influencing factors
HEI	the energy input per unit of star-rated hotels revenue		
TEI	the energy input per unit of travel agencies’ revenue	Subscripts	
SEI	the energy input per unit of scenic spots’ revenue	*th*	star-rated hotel
HDE	direct carbon emissions eco-efficiency of star-rated hotels	*ta*	travel agency
TDE	direct carbon emissions eco-efficiency of travel agencies	*ts*	scenic spot
SDE	direct carbon emissions eco-efficiency of scenic spots	*ac*	accommodation and catering sector
HTE	total carbon emissions eco-efficiency of star-rated hotels	*os*	other services sector
TTE	total carbon emissions eco-efficiency of travel agencies	*i*	the *i* th industry sector
STE	total carbon emissions eco-efficiency of scenic spots	*j*	the *j* th industry sector
PGDP	per GDP	*n*	the number of the industry sectors
THI	proportion of tertiary industry	*m*	the number of the regions
UR	proportion of urban population	*r*	the number of the types of fuel
ED	number of students in colleges and universities	*k*	the *k* th fuel
FR	total investment of foreign enterprises	*g*	the good output
RO	road mileage	*b*	the bad output
		*p*	the number of the input indicators
Notations		*q*	the years of eco-efficiency analysis
*X*	the total output matrix	*d*	the *d* th input indicator
*Y*	the final use matrix	*r*	the *r* th output indicator
*A*	the direct consumption coefficient matrix	*λ*	the intensity vector in SBM model
*I*	identity matrix	*t*	the *t* th year
*IS*	industrial sector	*direct*	direct carbon emissions
*L*	the value-added matrix	*total*	total carbon emissions
*P*	the production possibility set	*control*	control variables

## References

[1] UNWTO (2020). Annual Report 2019.

[2] Lee L.C., Wang Y., Zuo J. (2021). The nexus of water-energy-food in China’s tourism industry. Resour. Conserv. Recycl..

[3] Chenghu Z., Arif M., Shehzad K., Ahmad M., Oláh J. (2021). Modeling the Dynamic Linkage between Tourism Development, Technological Innovation, Urbanization and Environmental Quality: Provincial Data Analysis of China. Int. J. Environ. Res. Public Health.

[4] UNWTO (2010). Recommendations for Tourism Statistics 2008.

[5] Whittlesea E.R., Owen A. (2012). Towards a low carbon future—The development and application of REAP Tourism, a destination footprint and scenario tool. J. Sustain. Tour..

[6] Sun Y.-Y. (2014). A framework to account for the tourism carbon footprint at island destinations. Tour. Manag..

[7] Lenzen M., Sun Y.-Y., Faturay F., Ting Y.-P., Geschke A., Malik A. (2018). The carbon footprint of global tourism. Nat. Clim. Chang..

[8] Guan D., Wang D., Hallegatte S., Davis S.J., Huo J., Li S., Bai Y., Lei T., Xue Q., Coffman D. (2020). Global supply-chain effects of COVID-19 control measures. Nat. Hum. Behav..

[9] Sun Y.-Y., Higham J. (2021). Overcoming information asymmetry in tourism carbon management: The application of a new reporting architecture to Aotearoa New Zealand. Tour. Manag..

[10] Sun Y.-Y. (2018). Global Value Chains and National Tourism Carbon Competitiveness. J. Travel Res..

[11] Matveeva N.S., Nazarov V.S. (2021). Legislative Regulation Financial Statement Preparation by Micro Entities: International Experience. Financ. J..

[12] UNWTO (2017). Tourism and the Sustainable Development Goals—Journey to 2030.

[13] Paiano A., Crovella T., Lagioia G. (2019). Managing sustainable practices in cruise tourism: The assessment of carbon footprint and waste of water and beverage packaging. Tour. Manag..

[14] Sun Y.-Y., Drakeman D. (2020). Measuring the carbon footprint of wine tourism and cellar door sales. J. Clean. Prod..

[15] Ehrenfeld J.R. (2005). Eco-efficiency—Philosophy, theory, and tools. J. Ind. Ecol..

[16] Gössling S., Peeters P., Ceron J.-P., Dubois G., Patterson T., Richardson R. (2005). The eco-efficiency of tourism. Ecol. Econ..

[17] Sun Y.-Y., Lin P.-C., Higham J. (2020). Managing tourism emissions through optimizing the tourism demand mix: Concept and analysis. Tour. Manag..

[18] Papavasileiou E.F., Tzouvanas’ P. (2021). Tourism Carbon Kuznets-Curve Hypothesis: A Systematic Literature Review and a Paradigm Shift to a Corporation-Performance Perspective. J. Travel Res..

[19] Pan Y., Weng G., Li C., Li J. (2021). Coupling Coordination and Influencing Factors among Tourism Carbon Emission, Tourism Economic and Tourism Innovation. Int. J. Environ. Res. Public Health.

[20] Zha J., Tan T., Yuan W., Yang X., Zhu Y. (2019). Decomposition analysis of tourism CO_2_ emissions for sustainable development: A case study of China. Sustain. Dev..

[21] Ruan W., Li Y., Zhang S., Liu C.-H. (2019). Evaluation and drive mechanism of tourism ecological security based on the DPSIR-DEA model. Tour. Manag..

[22] Zha J., Zhu Y., He D., Tan T., Yang X. (2020). Sources of tourism growth in Mainland China: An extended data envelopment analysis-based decomposition analysis. Int. J. Tour. Res..

[23] Zhang J., Zhang Y. (2020). Assessing the low-carbon tourism in the tourism-based urban destinations. J. Clean. Prod..

[24] Robaina-Alves M., Moutinho V., Costa R. (2016). Change in energy-related CO_2_ (carbon dioxide) emissions in Portuguese tourism: A decomposition analysis from 2000 to 2008. J. Clean. Prod..

[25] Puig R., Kiliç E., Navarro A., Albertí J., Chacón L., Fullana-I-Palmer P. (2017). Inventory analysis and carbon footprint of coastland-hotel services: A Spanish case study. Sci. Total Environ..

[26] Lee J.W., Brahmasrene T. (2013). Investigating the influence of tourism on economic growth and carbon emissions: Evidence from panel analysis of the European Union. Tour. Manag..

[27] Bianco V. (2020). Analysis of electricity consumption in the tourism sector. A decomposition approach. J. Clean. Prod..

[28] Katircioglu S.T. (2014). International tourism, energy consumption, and environmental pollution: The case of Turkey. Renew. Sustain. Energy Rev..

[29] Pereira R.P.T., Ribeiro G.M., Filimonau V. (2016). The carbon footprint appraisal of local visitor travel in Brazil: A case of the Rio de Janeiro-São Paulo itinerary. J. Clean. Prod..

[30] Cadarso M.Á., Gomez N., López L.A., Tobarra M.Á. (2016). Calculating tourism’s carbon footprint: Measuring the impact of investments. J. Clean. Prod..

[31] Yang Y., Li W. (2019). The evolution of the ecological footprint and its relationship with the urban development of megacities in Western China: The case of Xi’an. J. Environ. Manag..

[32] Chen Q., Mao Y., Morrison A.M. (2021). Impacts of Environmental Regulations on Tourism Carbon Emissions. Int. J. Environ. Res. Public Health.

[33] Wang Z., Yang Y., Wang B. (2018). Carbon footprints and embodied CO_2_ transfers among provinces in China. Renew. Sustain. Energy Rev..

[34] Huang Z., Cao F., Jin C., Yu Z., Huang R. (2017). Carbon emission flow from self-driving tours and its spatial relationship with scenic spots—A traffic-related big data method. J. Clean. Prod..

[35] Tsai K.-T., Lin T.-P., Hwang R.-L., Huang Y.-J. (2014). Carbon dioxide emissions generated by energy consumption of hotels and homestay facilities in Taiwan. Tour. Manag..

[36] Luo F., Becken S., Zhong Y. (2018). Changing travel patterns in China and ‘carbon footprint’ implications for a domestic tourist destination. Tour. Manag..

[37] Mi Z., Zheng J., Meng J., Ou J., Hubacek K., Liu Z., Coffman D., Stern N., Liang S., Wei Y.-M. (2020). Economic development and converging household carbon footprints in China. Nat. Sustain..

[38] Xu Z., Li Y., Chau S.N., Dietz T., Li C., Wan L., Zhang J., Zhang L., Li Y., Chung M.G. (2020). Impacts of international trade on global sustainable development. Nat. Sustain..

[39] López L.A., Cadarso M.Á., Zafrilla J., Arce G. (2019). The carbon footprint of the U.S. multinationals’ foreign affiliates. Nat. Commun..

[40] Cheng H., Dong S., Li F., Yang Y., Li S., Li Y. (2018). Multiregional Input-Output Analysis of Spatial-Temporal Evolution Driving Force for Carbon Emissions Embodied in Interprovincial Trade and Optimization Policies: Case Study of Northeast Industrial District in China. Environ. Sci. Technol..

[41] Wiedmann T., Minx J., Pertsova C.C. (2008). Definition of Carbon Footprint. Ecological Economics Research Trends.

[42] Berners-Lee M., Howard D.C., Moss J., Kaivanto K., Scott W.A. (2011). Greenhouse gas footprinting for small businesses—The use of input–output data. Sci. Total Environ..

[43] Frampton S.C. (2003). Energy use associated with different travel choices. Tour. Manag..

[44] Kuo N.-W., Lin C.-Y., Chen P.-H., Chen Y.-W. (2009). An inventory of the energy use and carbon dioxide emissions from island tourism based on a life cycle assessment approach. Environ. Prog. Sustain. Energy.

[45] Gamboa M.M., Iribarren D., Dufour J. (2018). Environmental impact efficiency of natural gas combined cycle power plants: A combined life cycle assessment and dynamic data envelopment analysis approach. Sci. Total Environ..

[46] AbdelAzim A.I., Ibrahim A.M., Aboul-Zahab E.M. (2017). Development of an energy efficiency rating system for existing buildings using Analytic Hierarchy Process—The case of Egypt. Renew. Sustain. Energy Rev..

[47] Tsaples G., Papathanasiou J. (2020). Data envelopment analysis and the concept of sustainability: A review and analysis of the literature. Renew. Sustain. Energy Rev..

[48] Quintano C., Mazzocchi P., Rocca A. (2020). Examining eco-efficiency in the port sector via non-radial data envelopment analysis and the response based procedure for detecting unit segments. J. Clean. Prod..

[49] Gössling S., Higham J. (2020). The Low-Carbon Imperative: Destination Management under Urgent Climate Change. J. Travel Res..

[50] Xia B., Dong S., Ba D., Li Y., Li F., Liu H., Zhao M. (2018). Research on the Spatial Differentiation and Driving Factors of Tourism Enterprises’ Efficiency: Chinese Scenic Spots, Travel Agencies, and Hotels. Sustainability.

[51] Yang Y., Yao C., Xu D. (2020). Ecological compensation standards of national scenic spots in western China: A case study of Taibai Mountain. Tour. Manag..

[52] Wang L., Zhou X., Lu M., Cui Z. (2020). Impacts of haze weather on tourist arrivals and destination preference: Analysis based on Baidu Index of 73 scenic spots in Beijing, China. J. Clean. Prod..

[53] W. O. Economics (2017). 2017 Travel & Tourism Economic Impact Research.

[54] Koiwanit J., Filimonau V. (2021). Carbon footprint assessment of home-stays in Thailand Resource. Conserv. Recycl..

[55] Zhang F., Zhang M., Wang S., Qiang F., Che Y., Wang J. (2017). Evaluation of the tourism climate in the Hexi Corridor of northwest China’s Gansu Province during 1980–2012. Arch. Meteorol. Geophys. Bioclimatol. Ser. B.

[56] Zhang K., Liu X., Yao J. (2019). Spatial correlation between the agglomeration and CO_2_ emissions of China’s tourism industry. Resour. Sci..

[57] Rebolledo-Leiva R., Vásquez-Ibarra L., Entrena-Barbero E., Fernández M., Feijoo G., Moreira M.T., González-García S. (2022). Coupling Material Flow Analysis and Network DEA for the evaluation of eco-efficiency and circularity on dairy farms. Sustain. Prod. Consump..

[58] Li Y., Zuo Z., Xu D., Wei Y. (2021). Mining Eco-Efficiency Measurement and Driving Factors Identification Based on Meta-US-SBM in Guangxi Province, China. Int. J. Environ. Res. Public Health.

[59] Cooper W.W., Seiford L.M., Tone K. (2007). Data Envelopment Analysis. A Comprehensive Text with Models, Applications, References and DEA-Solver Software.

[60] McDonald J.F., Moffitt R.A. (1980). The Uses of Tobit Analysis. Rev. Econ. Stat..

[61] Cheng Y., Shao T., Lai H., Shen M., Li Y. (2019). Total-Factor Eco-Efficiency and Its Influencing Factors in the Yangtze River Delta Urban Agglomeration, China. Int. J. Environ. Res. Public Health.

[62] Chen Y., Yin G., Liu K. (2021). Regional differences in the industrial water use efficiency of China: The spatial spillover effect and relevant factors. Resour. Conserv. Recycl..

[63] Ren Y., Fang C., Li G. (2020). Spatiotemporal characteristics and influential factors of eco-efficiency in Chinese prefecture-level cities: A spatial panel econometric analysis. J. Clean. Prod..

[64] Zhang J., Wang S., Yang P., Fan F., Wang X. (2020). Analysis of Scale Factors on China’s Sustainable Development Efficiency Based on Three-Stage DEA and a Double Threshold Test. Sustainability.

[65] Zhou Y., Kong Y., Sha J., Wang H. (2019). The role of industrial structure upgrades in eco-efficiency evolution: Spatial correlation and spillover effects. Sci. Total Environ..

[66] He L., Zha J., Loo H.A. (2020). How to improve tourism energy efficiency to achieve sustainable tourism: Evidence from China. Curr. Issues Tour..

[67] Luo F., Moyle B.D., Moyle C.-L.J., Zhong Y., Shi S. (2020). Drivers of carbon emissions in China’s tourism industry. J. Sustain. Tour..

[68] Hu W., Guo Y., Tian J., Chen L. (2019). Eco-efficiency of centralized wastewater treatment plants in industrial parks: A slack-based data envelopment analysis. Resour. Conserv. Recycl..

[69] Tang M., Li Z., Hu F., Wu B. (2020). How does land urbanization promote urban eco-efficiency? The mediating effect of industrial structure advancement. J. Clean. Prod..

[70] Chen W., Ning S., Chen W., Liu E.-N., Wang Y., Zhao M. (2020). Spatial-temporal characteristics of industrial land green efficiency in China: Evidence from prefecture-level cities. Ecol. Indic..

[71] Wang J., Wang S., Li S., Cai Q., Gao S. (2019). Evaluating the energy-environment efficiency and its determinants in Guangdong using a slack-based measure with environmental undesirable outputs and panel data model. Sci. Total Environ..

[72] Arceo A., Biswas W.K., John M. (2019). Eco-efficiency improvement of Western Australian remote area power supply. J. Clean. Prod..

[73] Zha J., Yuan W., Dai J., Tan T., He L. (2020). Eco-efficiency, eco-productivity and tourism growth in China: A non-convex metafrontier DEA-based decomposition model. J. Sustain. Tour..

[74] Meng W.Q., Xu L.Y., Hu B.B., Zhou J., Wang Z.L. (2017). Quantifying direct and indirect carbon dioxide emissions of the Chinese tourism industry. J. Clean. Prod..

[75] Becken S., Patterson M. (2006). Measuring National Carbon Dioxide Emissions from Tourism as a Key Step Towards Achieving Sustainable Tourism. J. Sustain. Tour..

[76] Dwyer L., Forsyth P., Spurr R., Hoque S. (2010). Estimating the carbon footprint of Australian tourism. J. Sustain. Tour..

[77] Cheng Z., Li L., Liu J. (2018). Industrial structure, technical progress and carbon intensity in China’s provinces. Renew. Sust. Energ. Rev..

[78] Zaman K., Shahbaz M., Loganathan N., Raza S.A. (2016). Tourism development, energy consumption and Environmental Kuznets Curve: Trivariate analysis in the panel of developed and developing countries. Tour. Manag..

[79] Xue D., Yue L., Ahmad F., Draz M.U., Chandio A.A. (2021). Urban eco-efficiency and its influencing factors in Western China: Fresh evidence from Chinese cities based on the US-SBM. Ecol. Indic..

[80] Haibo C., Ke D., Fangfang W., Ayamba E.C. (2020). The spatial effect of tourism economic development on regional ecological efficiency. Environ. Sci. Pollut. Res..

[81] Lu C., Li W., Pang M., Xue B., Miao H. (2018). Quantifying the Economy-Environment Interactions in Tourism: Case of Gansu Province, China. Sustainability.

[82] Moiseev N., Mikhaylov A., Varyash I., Saqib A. (2020). Investigating the relation of GDP per capita and corruption index. Entrep. Sustain. Issues.

[83] Li Y., Filimonau V., Wang L.-E., Cheng S. (2019). Tourist food consumption and its arable land requirements in a popular tourist destination. Resour. Conserv. Recycl..

[84] Yi-Ming W., Meng J., Guan D., Shan Y., Song M., Wei Y.-M., Liu Z., Hubacek K. (2017). Chinese CO2 emission flows have reversed since the global financial crisis. Nat. Commun..

[85] Bushukina V.I. (2021). Specific Features of Renewable Energy Development in the World and Russia. Financ. J..

[86] Kranina E.I. (2021). China on the Way to Achieving Carbon Neutrality. Financ. J..

